# Efficient removal of noxious methylene blue and crystal violet dyes at neutral conditions by reusable montmorillonite/NiFe_2_O_4_@amine-functionalized chitosan composite

**DOI:** 10.1038/s41598-022-19570-1

**Published:** 2022-09-15

**Authors:** Hassanien Gomaa, Eman M. Abd El-Monaem, Abdelazeem S. Eltaweil, Ahmed M. Omer

**Affiliations:** 1 Department of Chemistry, Faculty of Science, Al-Azhar University, Assiut, 71524 Egypt; 2 Chemistry Department, Faculty of Science, Alexandria University, Alexandria, Egypt; 3 Polymer Materials Research Department, Advanced Technology and New Materials Research Institute (ATNMRI), City of Scientific Research and Technological Applications (SRTA-City), New Borg El-Arab City, Alexandria, 21934 Egypt

**Keywords:** Green chemistry, Inorganic chemistry, Materials science, Environmental chemistry

## Abstract

The jeopardy of the synthetic dyes effluents on human health and the environment has swiftly aggravated, threatening human survival. Hence, sustained studies have figured out the most acceptable way to eliminate this poisonous contaminant. Thereby, our investigation aimed to fabricate montmorillonite/magnetic NiFe_2_O_4_@amine-functionalized chitosan (MMT-mAmCs) composite as a promising green adsorbent to remove the cationic methylene blue (MB) and crystal violet (CV) dyes from the wastewater in neutral conditions. Interestingly, MMT-mAmCs composite carries high negative charges at a wide pH range from 4 to 11 as clarified from zeta potential measurements, asserting its suitability to adsorb the cationic contaminants. In addition, the experimental study confirmed that the optimum pH to adsorb both MB and CV was pH 7, inferring the ability of MMT-mAmCs to adsorb both cationic dyes in simple process conditions. Furthermore, the ferromagnetic behavior of the MMT-mAmCs composite is additional merit to our adsorbent that provides facile, fast, and flawless separation. Notably, the as-fabricated composite revealed an auspicious adsorbability towards the adsorptive removal of MB and CV, since the maximum adsorption capacity of MB and CV were 137 and 118 mg/g, respectively. Moreover, the isotherm and kinetic investigatins depicted that the adsorption of both cationic dyes fitted Langmuir and Pseudo 2nd order models, respectively. Besides, the advanced adsorbent preserved satisfactory adsorption characteristics with maximal removal efficacy exceeding 87% after reuse for ten consecutive cycles. More importantly, MMT-mAmCs efficiently adsorbed MB and CV from real agricultural water, Nile river water and wastewater samples at the neutral pH medium, reflecting its potentiality to be a superb reusable candidate for adsorptive removal cationic pollutants from their aquatic media.

## Introduction

Certainly, the broad consumption of synthetic dyes in industries such as textiles, papers, pharmaceuticals, printing, food, etc., is causing great jeopardy to life on Earth^1–3^. The harmful impacts of these detrimental dyes are not limited to humans, but also wasteful dyeing process generates colored wastewater that pollutes the environment and affects the aquatic ecosystems^4–6^. Among the notorious cationic dyes, methylene blue (MB) and crystal violet (CV) possess complicated structures, rendering their removal from wastewater a big issue^7–9^. Both have significant threats to human health. MB dye may lead to heartbeat upsurge, tissue necrosis, vomiting, shock, chest hurt, and gastritis^10^. Similarly, CV dye can be absorbed through inhalation and ingestion and the skin, leading to horrible irritation, acute eye inflammation, breathing difficulty, nausea, hypertension, painful sensitization, and kidney failure^11,12^. Despite all these harmful impacts, these carcinogenic dyes are continually produced and consumed commercially^13^. Thus, governments and authorities have been forced to introduce strict legislation on the permissible concentration of colored dye contaminants released by industries^13,14^.

Commonly, industrial wastewater is treated by numerous conventional techniques for eliminating the synthetic dyes from wastewater, including adsorption, chemical degradation, chemical coagulation, precipitation, advanced oxidation, and biological treatments^15–20^. Among these techniques, the adsorption method has been elaborated to meet the required standards for efficient removal of toxic dyes due to its simple, eco-friendly, and high performance^21,22^.

Recently, research interest has been diverted towards naturally occurring bio-polymers-based adsorbents such as cellulose, chitosan, alginate, and starch due to their unique characteristics, for example, non-toxic, low-priced production, biodegradability, and good adsorption properties^23–25^. Chitosan (Cs) is a marine biopolymer produced by simple deacetylation of chitin biopolymer that is considered the major ingredient of the exoskeleton of crustacean shells^26–28^. Due to its appealing features such as lack of toxicity, availability in nature, biocompatibility, biodegradability, and ease of modification, Cs has been expansively employed as an efficient candidate in various biomedical, industrial, and water treatment applications^29^. In addition, Cs has been effectually applied to removing multiple contaminants from their aquatic media, such as dyes^30^, pharmaceutical residues^31,32^, and heavy metals^33^. Nevertheless, pure Cs possess serious shortcomings: low adsorption capacity, limited surface area, and poor mechanical properties^34^. To overcome these shortcomings, various physicochemical modifications have been performed to pristine Cs, such as grafting, Schiff base formation and crosslinking, and composite formation with clays, carbon based-materials, and MOFs materials^35,36^. For instance, amine-functionalized Cs is a freshly modified Cs derivative with more NH_2_ groups, which is supposed to enhance the adsorption properties of Cs^37^.

Montmorillonite (MMT) is a naturally occurring clay mineral that comprises sheets of two tetrahedral silica (SiO_2_) layers sandwiching one octahedral alumina (Al_2_O_3_) layer^38^. MMT clay demonstrates tangible advantages, including eco-friendly, low-cost production, high cation exchange capacity, and a higher surface area with its layered structure^39^. Thus, MMT has been used to remove various cationic and anionic dyes^40^. Interestingly, as promising adsorbents, Clay/Polymer composites have drawn much interest, having unappalled advantages, including high mechanical properties, auspicious adsorption performance, and high surface area^41^. Nonetheless, such composites suffer poor reusability; thereby, incorporating a magnetic material into the Clay/Polymer skeleton is practicable to ameliorate its reusability and adsorbability^42^.

Nickel ferrite (NiFe_2_O_4_), as cubic ferromagnetic oxide, is one of the most versatile ferrites with typical inverse spinel structure^43^. It has prodigious merits such as exceptional magnetic and electromagnetic features, super chemical and mechanical stability and low conductivity^44^. Accordingly, NiFe_2_O_4_ particles have been utilized in a wide-scale as adsorbent^45^, photocatalytic degradation^46^, biosensors^47^ and medicine^48^. Such special characteristics have acquired NiFe_2_O_4_ fame in the wastewater remediation field as a promising adsorbent with easy separation merit^49^. Therefore, NiFe_2_O_4_ has been effectively employed as a powerful magnetic candidate for providing ease-separation process in addition to enhancing the mechanical stability and reusability of various magnetic adsorbent composites^50,51^.

To satisfy the needs of high adsorption performance, respectable regeneration and ease of separation, we aimed in this study to combine the individual adsorption features of chitosan derivative, NiFe_2_O_4_ and MMT clay through magnetic composite formation. The as-fabricated green MMT-mAmCs composite was employed as adsorbent candidate for efficient removal of CV and MB dyes at a neutral medium. The developed magnetic composite was characterized using several characterization tools. In addition, several batch-sorption trials thoroughly investigated factors impacting the adsorption process. Additionally, isotherms, kinetics, and thermodynamic investigations were presented. The aptitude of MMT-mAmCs composite for regeneration and reuse for several cycles was also evaluated. Besides, the actual applicability of the developed adsorbent was examined using real dirty water models.

## Experimental

### Materials

Montmorillonite K-10 (MMT, S_BET_ = 240 m^2^g^−1^) was obtained from Aladdin Industrial Co. (Shanghai-China). p-Benzoquinone PBQ (98%), chitin, and ethylenediamine EDA (99%) were supplied by Sigma-Aldrich. NaOH (99%), *N,N*-dimethyl formamide (99.5%), and absolute ethanol (99.8%) were obtained from Alpha Aesar. HCl (12 M), NH_4_OH (25%), and CH_3_COOH (98%) were bought from Aladdin Reagent Co., Ltd. Analytical grade NiCl_2_.6H_2_O and FeCl_3_.6H_2_O were supplied by MP Biomedicals, LLC.

### Characterization tools

Fourier transform infrared spectra were used to identify the main functional groups in the prepared samples (FTIR; Tensor-II, Bruker, Germany) at a wavenumber range of 4000–400 cm^−1^. The thermal behaviour of the samples was investigated using thermo-gravimetric analysis (TGA; Perkin-Elmer, USA) with a heating rate of 10 °C/ min under a nitrogen flow rate about 20 mL/min. Morphological characterization was explored using scanning electron microscope (SEM; S4800-Hitachi, Japan). The analyzed sample was prepared as follows; 5 mg of sample was dispersed in 10 mL ethanol for 10 h under sonication. Then, few drops of the resultant suspension were put onto grid coated with copper. The magnetic behaviour of the samples was examined by vibrating-sample magnetometer (VSM; Lake shore; USA), while X-ray Photoelectron Spectroscopy was performed using (XPS; Thermo-Fisher Sci., USA) in the range of 20–1300 eV. The surface charge of MMT-mAmCs was determined utilizing Zeta-sizer (ZP; Malvern, UK). The examined sample was prepared as follows; preparing a diluted suspension of sample, adjusting the suspension pH at the range of 1–11 and sonicating the suspension for 2 h. The crystal profile was investigated by X-ray diffractometer (XRD; M03XHF, MAC Science, Japan) with wavelength λ = 1.54 A° (Cu–Kα), at a tube voltage of 35 kV and tube current of 30 mA.

### Preparation of AmCs

The AmCs was prepared for our previous work with slight modifications^44^. Firstly, chitin (7 g) was immersed into a solution of PBQ (6.8 mM, 100 mL) at pH 9, under mixing at 60 °C for 6 h. The obtained solid was then filtered and rinsed with dist. H_2_O to give PBQ-chitin which was soaked in EDA aqueous solution (6.9 mM, 100 mL) with constant stirring at 60 °C for 6 h. The acquired product was centrifuged and rinsed with dist. H_2_O to eliminate the extra EDA to give aminated chitin. The obtained aminated chitin was subjected to deacetylation by stirring in NaOH (50%) at 130 °C for 24 h. The formed AmCs was centrifuged and washed with dist. H_2_O till pH reached 7, then dried overnight at 50 °C.

### Preparation of NiFe_2_O_4_

NiFe_2_O_4_ NPs were prepared via the co-precipitation method in which NiCl_2_.6H_2_O and FeCl_3_.6H_2_O were dissolved in 50 mL H_2_O the molar ratio 1:2, respectively. To this solution, 10 mL glycerol was added dropwise with continuous stirring for one h. After that, aqueous NaOH (12 M) solution was added slowly to the mixture till pH 12, followed by raising the temperature up to 85 °C with continuous stirring for an additional 2 h. The formed black suspension was moved to an autoclave and kept at 120 °C for 16 h. Lastly, the obtained NiFe_2_O_4_ NPs was rinsed repeatedly by dist. H_2_O till pH 7 and then dried at 80 °C in the oven overnight.

### Preparation of MMT-mAmCs

**Scheme 1:**
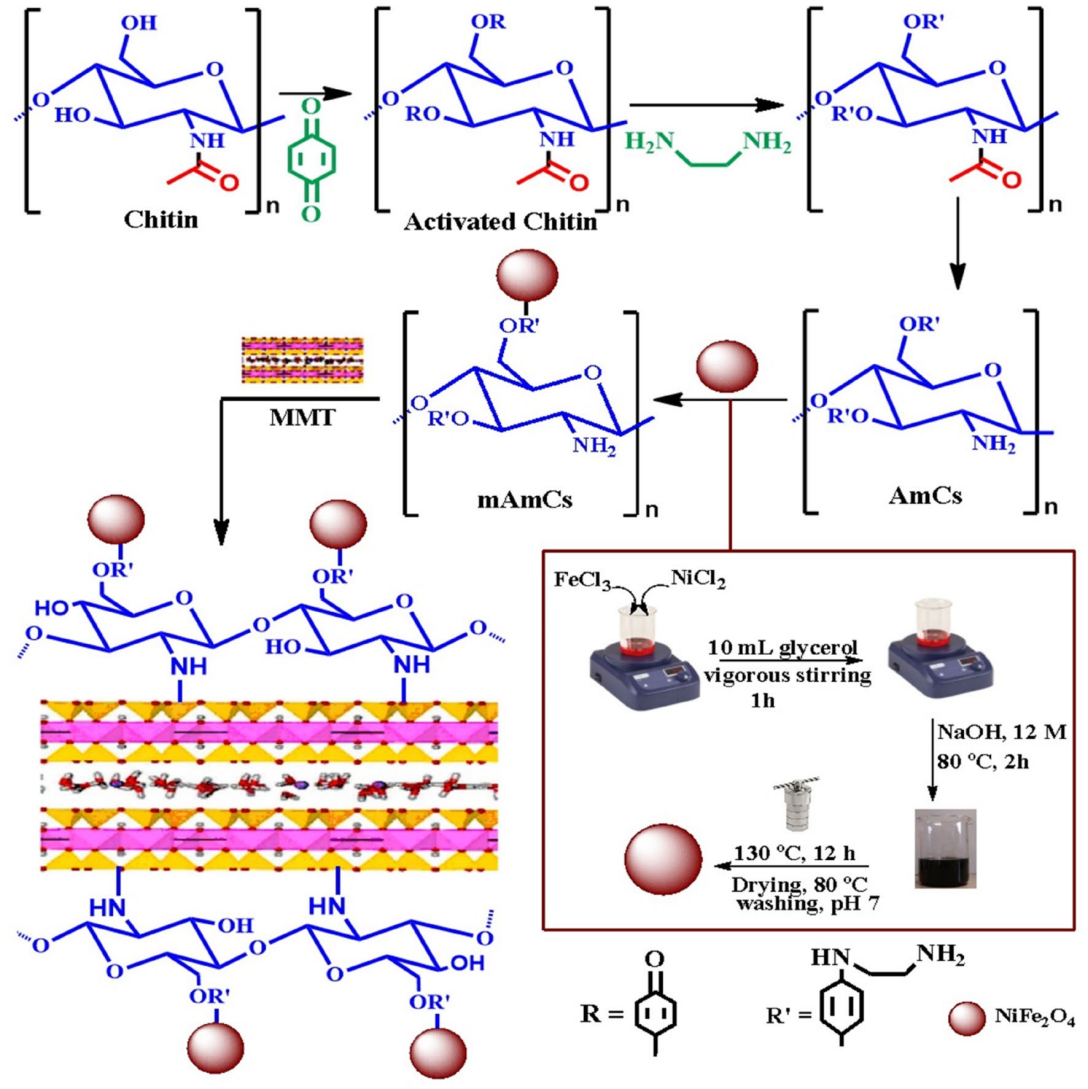
Schematic representation of MMT-mAmCs composite fabrication.

A certain amount of AmCs was dissolved in 25 mL of acetic acid (2%; v/v) solution and sonicated for 45 min. Then, 0.02 g of NiFe_2_O_4_ was inserted into the AmCs solution under stirring for 2 h to acquire an identical mixture. To the obtained homogenous mixture, a certain amount of MMT clay was added, followed by the addition of 4 mL of 25% glutaraldehyde, and then the mixture was stirred for 90 min at 60 °C. After all, the MMT-mAmCs composite was separated, washed with absolute ethanol, and then dried in an oven at 45 °C. Three different ratios of MMT:mAmCs were prepared as follow; MMT-mAmCs (1:3), MMT-mAmCs (1:1) and MMT-mAmCs (3:1), respectively. A chart representation for the MMT-mAmCs composite fabrication is shown in Scheme 1.

### Batch experiments

The current manuscript is designed to treat two organic dyes, such as MB and CV, through an easy and economical method using MMT-mAmCs to produce MB/CV-free water for human use. A batch-system procedure was applied for the adsorption investigations to achieve dye-removal data. Stock solutions (1000 ppm) of MB and CV were made by dissolving 1 g of MB and CV into 1 L double distilled water, then stored in dark bottles. The desired concentrations of MB and CV were prepared by dilution by double distilled water. In a typical experiment, 50 mL of MB/CV solution was agitated with 50 mg of MMT-mAmCs at an optimum pH, time, and temperature. pH values of solutions were adjusted in the batch operation experiment by adding small amounts of 0.1 M solutions of NaOH and HCl. The effects of pH, stirring time, MB/CV concentration, and temperatures on the MB and CV adsorption efficiency and capacity were systematically investigated to achieve optimum dye-removal conditions. MMT-mAmCs was filtered from equilibrated MMT-mAmCs–dye solution via centrifugation (5000 rpm). The MB and CV concentrations were assessed via UV–Vis spectrophotometer (Evolution 300, Thermo Scientific, England) at λ_max_ = 658, and 580 nm, respectively. The MB/CV removal efficiency % and the adsorption capacity of the MMT-mAmCs (q_e_, mg/g) were estimated through the next equivalences^12^:

1 $$ Dye - removal \% = \frac{{\left( {C_{i} - C_{f} } \right)}}{{C_{i} }} \times 100 $$

2 $$ q_{e} = \left( {C_{i} - C_{f} } \right)\left( \frac{V}{w} \right) $$

C_i_ and C_f_ are MB and CV dyes’ initial and final concentrations (i.e., before and after the removal process). V is the MB/CV solution volume of (L), and w is the MMT-mAmCs (g) quantity. To assess the performance of the MMT-mAmCs in removing MB and CV from aqueous solution and understanding the possible adsorption mechanism, isotherm, kinetic and thermodynamic investigations have been investigated. The regeneration of the used MMT-mAmCs was performed according to the elution treatment protocol using HNO_3_ as eluent agents. The regenerated MMT-mAmCs was reused for several repeated cycles through the batch-system technique. The real applicability of MMT-mAmCs was done by removing MB and CV dyes from real water samples.

## Results and discussion

### Fabrication mechanism

The fabrication mechanism of MMT-mAmCs composite was divided into three consecutive stages as delobrated in Scheme 1. The first stage involves the activation of –OH groups of chitin biopolymer under alkaline conditions using PBQ, which act as an activator. The reason for the activation process is to create active sites on the surface of chitin which facilitate its chemical modification. Next, EDA molecules were easily reacted with the activated –OH groups to produce amine-functionalized chitin, which followed by deacetylation process to convert the acetyl groups (–NHCOCH_3_) to the primary active NH_2_ groups. The second stage includes combination of AmCs with NiFe_2_O_4_ through various electrosatatic and chelation interactions between the generated extra positively charged –NH_2_ groups of AmCs with the negatively charged NiFe_2_O_4_. The third stage involves the reaction of –OH and –NH_2_ groups of mAmCs with Si–O and –OH groups on MMT clay surface through hydrogen bonding interactions.

### Characterization of MMT-mAmCs

#### FTIR

**Figure 1:**
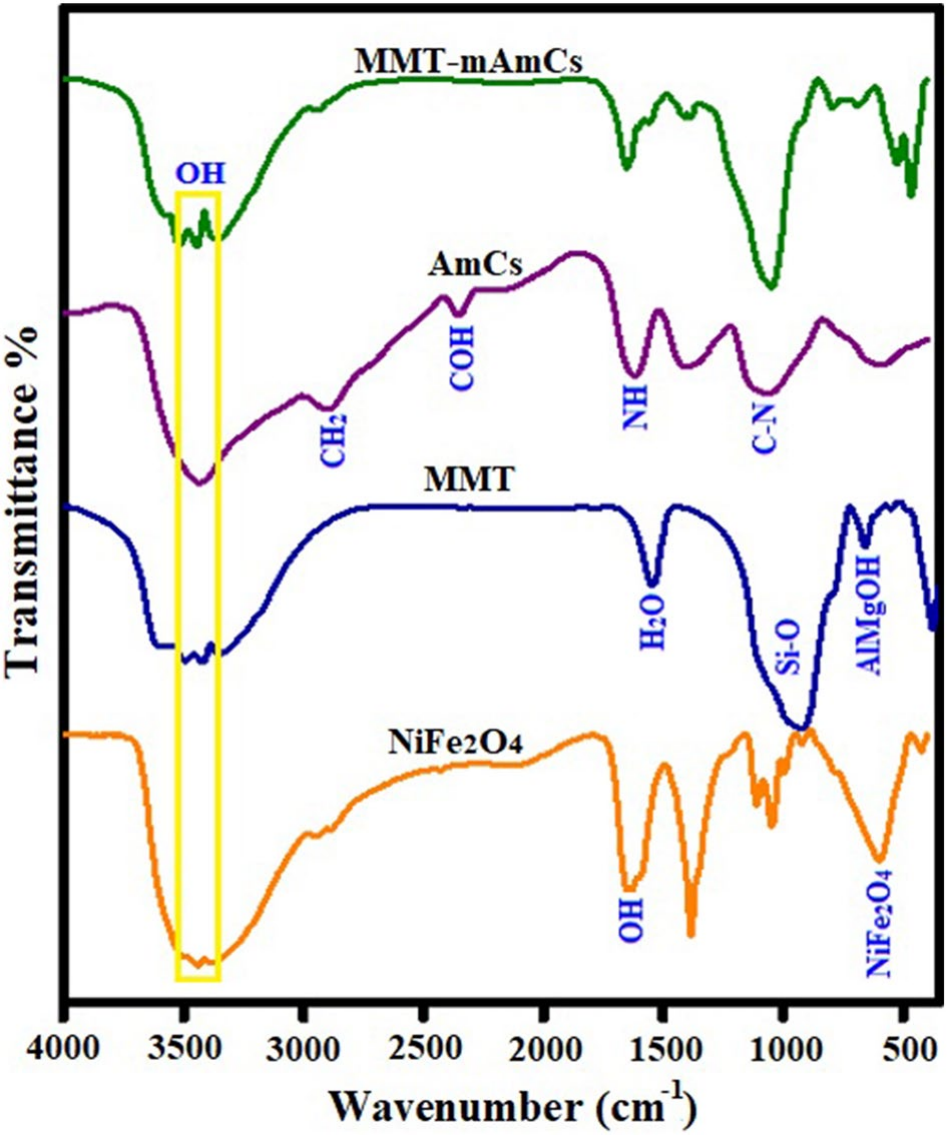
FTIR of NiFe_2_O_4_, MMT, AmCs and MMT-mAmCs composite.

Figure 1 represents the FTIR spectra of NiFe_2_O_4_, MMT, AmCs, and MMT-mAmCs composite. The FTIR curve of NiFe_2_O_4_ illustrates the absorbance peaks at 604 and 425 cm^−1^, suggesting the octahedral and tetrahedral modes of NiFe_2_O_4_, respectively. In addition, the characteristic peaks of OH appeared at 1641, 2935, and 3435 cm^−1^. The FTIR curve of MMT shows peaks at 804 and 1016 cm^−1^, which are ascribed to AlMgOH bending and Si–O stretching out of a plane, respectively^52^. The absorbance peaks at 3464 and 3516 cm^−1^ are corresponded to OH vibration of silicate and silanol groups, respectively, and the peak at 1662 cm^−1^ is attributed to H_2_O^53^. Moreover, the FTIR diagram of AmCs depicts absorption peaks at 1619, 2216, and 2901 cm^−1^. These peaks are assigned to NH bending vibrations, COH stretching, and CH_2_. In addition, the belonging peaks to –OH and C–N groups manifested at 3441 and 1062 cm^−1^, respectively. Furthermore, the transmittance peaks at 1402 and 2907 cm^−1^ are assigned to C–H in-plane bending and stretching vibrations, respectively. The FTIR spectrum of MMT-mAmCs evinces the successful blinding between MMT, NiFe_2_O_4,_ and AmCs since their discriminative absorbance peaks appeared obviously.

#### TGA

TGA is employed to examine substances’ thermal stability and thermal degradation behavior^54^. Figure S1 depicts the TGA profiles of NiFe_2_O_4_, MMT, AmCs, and MMT-mAmCs composite. The TGA profile of NiFe_2_O_4_ signalizes three weight loss stages; the first one is between 37 and 190 °C, attributed to solvent evaporation. In comparison, the second weight loss is between 190 and 229 °C, most likely due to the oxidation of the organic materials^55^. In addition, the weight loss between 229 and 696 °C is due to the corresponding metal oxide growth^56^. The TGA profile of MMT clarified the weight loss between 36 and 263 °C, which is assigned to the moisture removal and the dehydration of the hydrated cation. Furthermore, the slight weight loss after 263 °C is due to MMT’s de-hydroxylation. Moreover, the TGA profile of AmCs shows a mass deficiency between 36 and 150 °C, corresponding to water evaporation. In addition, the weight loss is caused by the dehydration of the saccharide rings, and de-polymerization of AmCs occurs between 150 and 350 °C. After 600 °C occurred, complete decomposition of the AmCs skeleton. TGA profile of MMT-mAmCs composite reveals a significant amelioration in the thermal behavior of AmCs, reflecting the advantage of incorporating such clay and magnetic material into the AmCs matrix.

#### VSM

**Figure 2:**
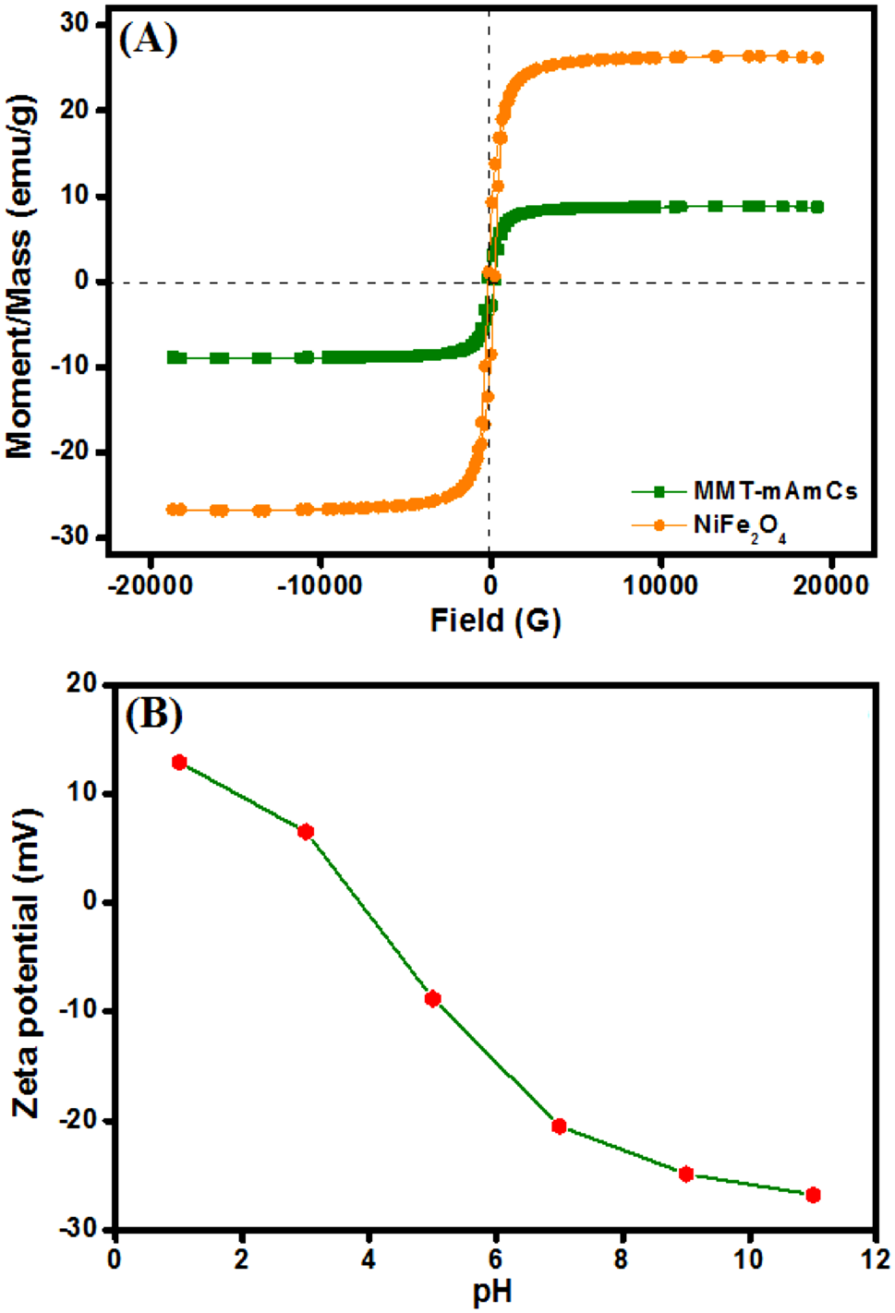
(**A**) VSM of NiFe_2_O_4_ and MMT-mAmCs composite and (**B**) ZP measurements of MMT-mAmCs composite.

It was found from VSM measurements (Fig. 2A) that the coercivity values of NiFe_2_O_4_ and MMT-mAmCs were 186.24 and 84.36 G, respectively, suggesting the ferromagnetic magnetic behaviors. Furthermore, a decline in the saturation magnetization (MS) of NiFe2O4 after blinding with MMT and AmCS since Ms of NiFe_2_O_4_ and MMT-mAmCs were 26.18 8.71 emu/g, respectively. Such a reduction in the Ms of NiFe_2_O_4_ may be attributed to the masking of polymer and clay layers. Nevertheless, the Ms value of MMT-mAmCs can still provide a perfect and facile separation after the adsorption process by an external magnet.

#### Zeta potential

ZP measurements (Fig. 2B) infer the suitability of the MMT-mAmCs composite to remove the cationic pollutants in neutral conditions since the point of zero charges was − 20.5 mV at pH 7. This finding endows one more advantage to the as-fabricated composite since it could naturally grasp the cationic contaminants from wastewater via the electrostatic interaction with superb efficiency without further adjustment to the pH medium. Hence, the as-fabricated MMT-mAmCs composite could provide an efficient and straightforward adsorption process to cationic dyes such as MB and CV.

#### SEM

**Figure 3:**
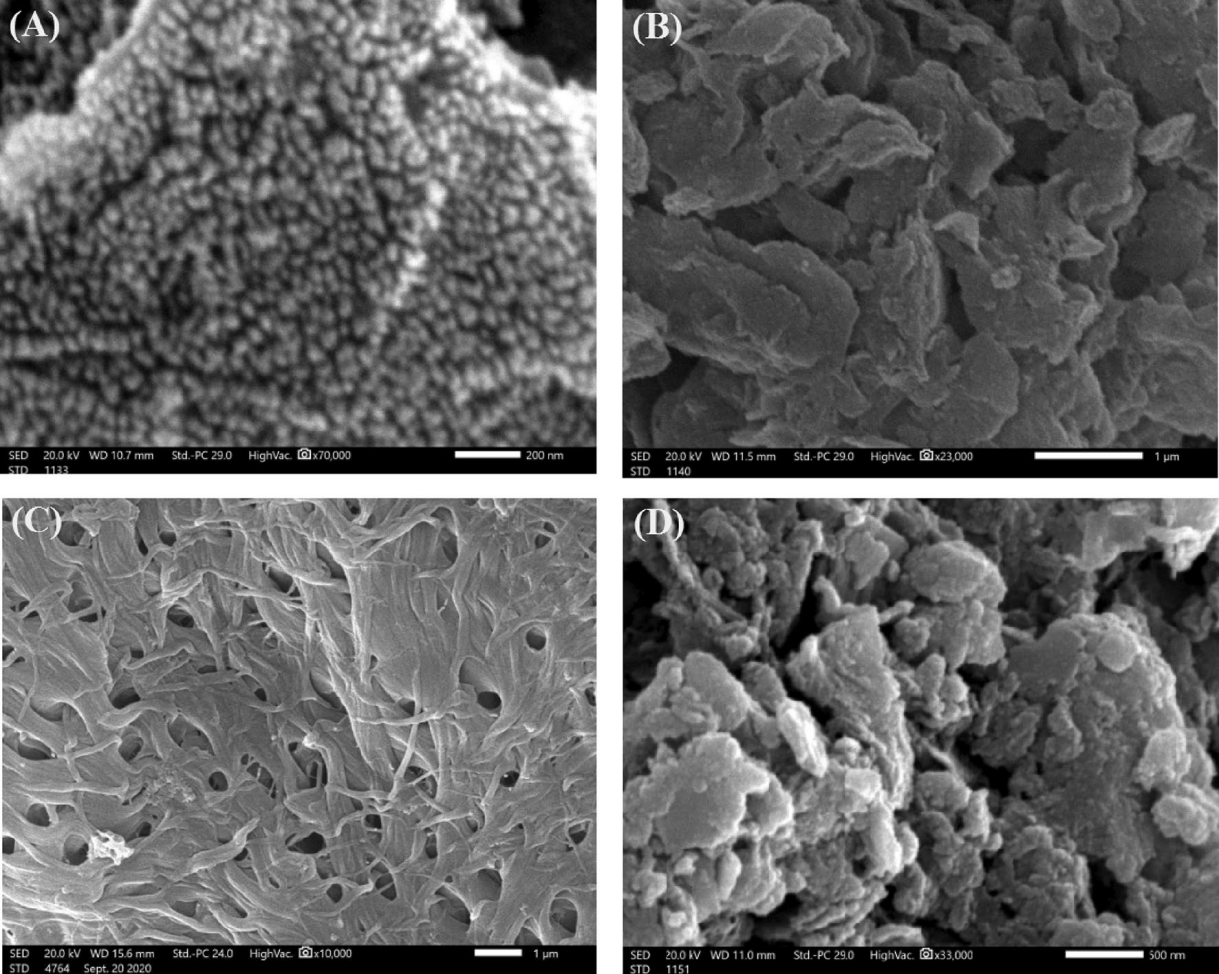
SEM images of (**A**) NiFe_2_O_4_, (**B**) MMT, (**C**) AmCs and (**D**) MMT-mAmCs composite.

The SEM image (Fig. 3A) shows aggregated nanoparticles of NiFe_2_O_4_, reflecting the excellent magnetic property of NiFe_2_O_4_. Furthermore, the SEM image (Fig. 3B) depicts accumulated large particles of lamellar MMT, while the surface morphology of AmCs looks like an interconnected network of porous structures (Fig. 3C). The SEM of the MMT-mAmCs composite (Fig. 3D) reveals the dispersed NiFe_2_O_4_ onto the irregular particles of the MMT-AmCs composite. Such morphology suggests the promising ability of MMT-mAmCs to adsorb the targeted contaminants owing to the plenty of active sites on its surface and its porous nature.

#### XPS

**Figure 4:**
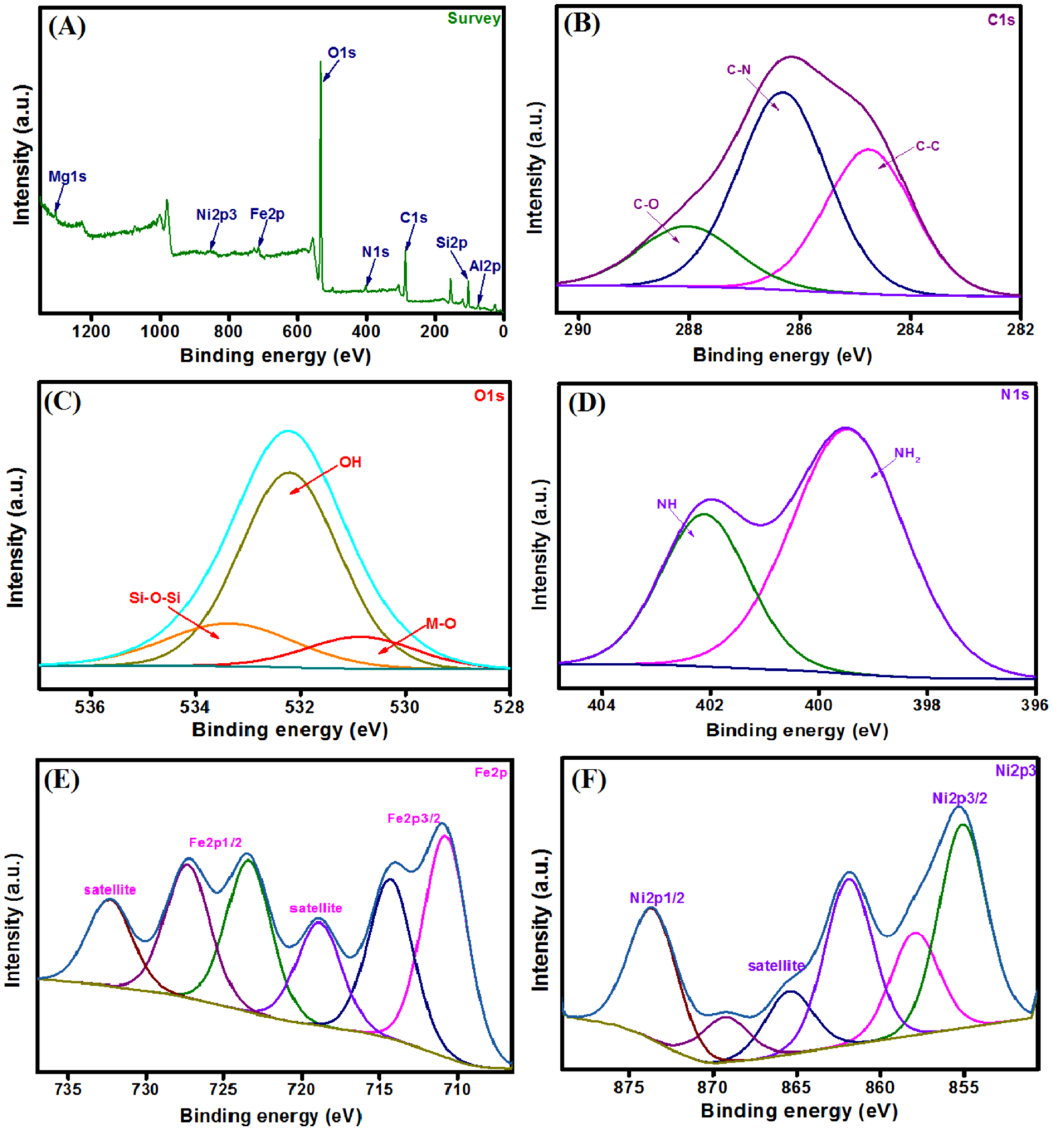
XPS spectra of MMT-mAmCS composite; (**A**) survey, (**B**) C1s, (**C**) O1s, (**D**) N1s, (**E**) Fe2p and (**F**) Ni2p3.

The XPS survey (Fig. 4A) signalizes the elemental composition of MMT-mAmCs composite; Al2p, Si2p, Mg1s, C1s, N1s, O1s, Fe_2_p and Ni2p3 at the binding energy (BE) of 75.37, 104.09, 1304.63, 287.11, 410.79, 533.39, 714.07 and 854.99 eV. Moreover, the C1s curve (Fig. 4B) figures out the peaks at 288.04, 284.75 and 286.31, which are attributed to C–O, C–C and C–N, respectively. The O1s curve (Fig. 4C) depicts the belonging peaks to Si–O–Si, M–O (Viz., Mg–O, Al–O, Ni–O and Fe–O) and OH groups at BE of 533.35, 530.84 and 532.21 eV, respectively. In addition, the N1s spectrum (Fig. 4D) represents the N– containing functional groups since the related peaks to NH and NH_2_ appeared at BE of 402.11 and 399.47 eV, respectively. The Fe2p spectrum (Fig. 4E) implies the presence of Fe^2+^ and Fe^3+^ since the characteristic peaks of Fe^2+^ manifested at BE of 710.78 and 723.41 eV, while the distinguishing peaks of Fe^3+^ at BE of 714.29 and 727.33 eV. The Ni2p3 spectrum (Fig. 4F) elucidates the belonging peaks to Ni2p3/2 at BE of 855.10, 857.95 and 861.90 eV as well as the peaks of N12p1/2 at BE of 873.59 eV.

### Optimization of MB and CV adsorption

This study mainly explains how the adsorption effectiveness of MB and CV dyes is affected by the parameters mentioned above. The batch methodology was employed to attain the efficient purpose of MMT-mAmCs in terms of the removal and adsorption of MB and CV dyes. Here, bench-top trials were completed to adjust the adsorption conditions, such as pH, stirring time, MMT-mAmCs dose, and dye-initial concentration.

#### Effect of pH and adsorption mechanism

**Figure 5:**
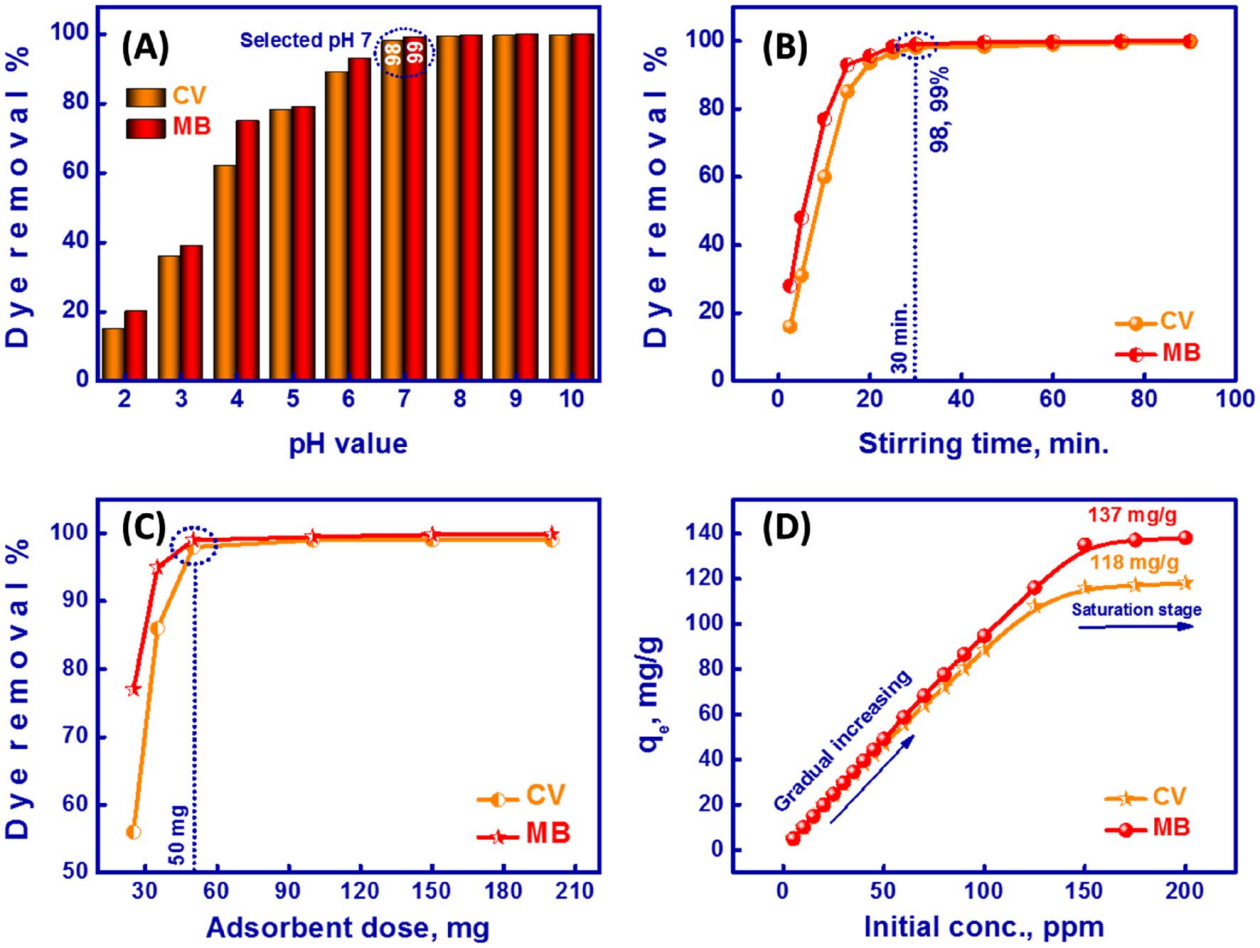
(**A**) Effect of pH, (**B**) Stirring time, (**C**) MMT-mAmCs dosage and (**D**) MB/CV concentration on the-removal % and capacity using MMT-mAmCs.

One of the key parameters that can be applied to remove MB and CV is the pH of a solution. pH is the greatest aspect influencing the charge type of MMT-mAmCs surface-active sites, causing either the boost or diminution of dye-removal effectiveness. In bench-top trials, 50 mg of MMT-mAmCs was agitated with 50 mL of MB/CV solution (25 ppm) at diverse pH conditions (pH 2–10). Figure 5A indicated that the MB/CV removal % was enhanced with pH growth. At pH 7, the MB and CV removal % were 99 and 98%, respectively. MB and CV dyes are cationic dyes and can be ionized into Cl^-^ ions and dye^+^ ions (i.e., positively MB^+^ and CV^+^ ions). Thus, both MB and CV exist as MB^+^ and CV^+^ ions at pH > 7^20,57^.

At pH < 7, active exterior sites of MMT-mAmCs become positively charged sites. These positively functional positions decrease the attraction of MB^+^ and CV^+^ ions onto MMT-mAmCs surface, thus decreasing the dye removal efficiency. The smaller removal % at pH < 7 may be due to the rivalry between H^+^ ions and MB^+^ and CV^+^ ions at surface-active sites^12^. At pH ≥ 7, the MMT-mAmCs surface is changed to negative positions as verified by ZP measurements, which boosts the adsorption of cationic MB and CV dyes. Thus, the removal of MB and CV dyes using MMT-mAmCs was found favorable at the higher pH values. Attributable to the insignificant variation between the removal % at pH 7 and pH > 7, pH 7 was selected as an optimal pH condition for the next investigations. The extreme removal % of the MMT-mAmCs at pH 7 may be owing to the following; (i) a huge quantity of surface-active positions interior/exterior grooves/pores and outside surface of the MMT-mAmCs adsorbent, and (ii) rapidity diffusion of MB and CV through the grooves/pores along with the MMT-mAmCs matrix. Hence, the adsorbent surface would participate in cation attraction and exchange reactions and the extraordinary electrostatic attraction between the cationic dye and the anionic adsorbent sites. Accordingly, the barrier to MB and CV molecules diffusion decreases, resulting in a high adsorption capacity. Table 1 shows the comparison between MMT-mAmCs adsorbent and other adsorbents used to remove MB and CV from wastewater, which were reported elsewhere, based on adsorption capacity (mg/g), and adsorption conditions. The obtained comparison indicated that MMT-mAmCs composite has a highly efficient and adsorption capacity toward MB and CV dyes due to the fast diffusion of dye molecules along composite’s grooves/pores.

**Table 1:** Comparison between MMT-mAmCs composite and other adsorbents reported elsewhere, in terms of adsorption capacity (mg/g), and adsorption conditions toward MB&CV removal.

Material name	Adsorption capacity, mg/g		pH conditions	Ref
	MB	CV		
MMT-mAmCs	137	118	7	Here
Sn/Si mixtures	30.13	36.83	6	^58^
Magnetic iron oxide/activated sericite nanocomposites	35.36	35.45	~ 6	^59^
Almond shell-based material	–	12.2	6	^60^
Lemongrass leaf fibers incorporated with cellulose acetate	–	36.10	7.47	^61^
Eco-Friendly Reduced Graphene Oxide	121.95	–	7	^62^
Mesoporous Ni–C–N/Silica aerogel	54	–	10	^63^
Chitosan–montmorillonite/polyaniline nanocomposite	111	–	–	^64^
Biochar from lychee seed	124.5	–	7	^65^
Modified rice husk	–	90.02	10	^66^
AgTiO_2_ Loading into Poly(3-Nitrothiophene)	–	43.10	8	^67^
β-cyclodextrin carbon-sphere-based nanocomposite	–	87.873	7.5	^68^
Copolymer adsorbent of AA and AMP	–	9.8	10	^69^

#### Effect of stirring period

The influence of the stirring period on the MB/CV removal % was also investigated because stirring time is substantial in the binding of MB and CV dyes with MMT-mAmCs active sites and thus the dye-adsorption capacity. Bench-top trials were done by whisking 50 mg of the MMT-mAmCs with 50 mL of MB and CV solution (25 ppm) for 2.5–90 min at pH 7. Finding results exhibited that the highest removal % can be attained within 30 min. Figure 5B indicated that the MMT-mAmCs adsorbent adsorbs more than 99% and 98% of MB and CV dyes, respectively. The findings explained that the removal of MB and CV was relatively fast at the early stages of the removal procedure and slackened before reaching the stability point^70^. This result confirmed that removing MB and CV is a rapid and time-dependent process. The low efficiency at stirring time < 30 min may be caused by inadequate contact time between MB&CV dyes and the MMT-mAmCs and the controlled diffusion of the MB&CV fragments from the bulk solution to the surface-active sites of the MMT-mAmCs adsorbent. Growing the stirring time enhances the MB&CV-removal %, which is considered an acceptable outcome for the real elimination of MB and CV dyes from actual wastewater models.

#### Effect of MMT-mAmCs dosage

The dose of MMT-mAmCs can influence the removal % of MB and CV dyes. The MMT-mAmCs quantity was adjusted from 25 to 200 mg at pH 7, room temperature, and 25 ppm MB/CV concentration. As revealed in Fig. 5C, the dye-removal % was improved with an expanded MMT-mAmCs dose. At smaller MMT-mAmCs quantities, the dye-adsorption % was insignificant due to the deficiency of surface-active sites^71^. In this case, 50 mg of the MMT-mAmCs has the maximum dye-removal ability (> 99 and 98% for both MB and CV). Therefore, future expanded the MMT-mAmCs amount is unnecessary, where the removal % remained almost constant. Hence, 50 mg of the MMT-mAmCs was enough to remove MB&CV dyes.

#### Effect of MB&CV concentration

The preliminary MB&CV concentration plays a significant role in offering a guiding force to conquer the unwillingness of mass gradient between the dye solution and the solid MMT-mAmCs adsorbent. Therefore, bench-top trials were performed by varying preliminary concentrations (5–200 ppm) to evaluate the maximum adsorption capability (q_e_, mg/g) of the proposed MMT-mAmCs. At the same time, the rest factors remain constant such as MMT-mAmCs dose (50 mg), pH of the solution (pH 7), stirring time (30 min), and temperature (room temp.). The result in Fig. 5D suggested that the adsorbed amount of MB and CV increased with the growth of dye concentration until it reached the equilibrium stage and maximum saturation capacity (137 and 118 mg/g for both MB and CV). At higher dye concentrations, the dye adsorption showed a slight increase due to competition of a huge quantity of MB and CV molecules at the active sites of the MMT-mAmCs adsorbent^72^.

### MB/CV-removal isotherm study

To explore the equilibrium between the MMT-mAmCs surface-active sites and the MB and CV in aqueous phases during the adsorption process, two commonly isotherm versions (Langmuir and Freundlich) are employed in the current manuscript. Freundlich isotherm clarifies the adsorption nature of heterogeneous systems in multilayer physical interaction by studying the relation between qe and Ce, which displays the heterogeneous nature of the MMT-mAmCs-surface. Freundlich equation provides info regarding the equilibrium phase between the MB&CV and MMT-mAmCs adsorbent. Langmuir’s model defines the adsorption nature of homogenous systems in monolayer as chemically interaction. The isotherm study for removing MB&CV dyes by MMT-mAmCs was performed under optimum sorption circumstances at different MB&CV concentrations varying from 5 to 200 ppm. Here, the essence of the MB&CV interaction with the MMT-mAmCs at the equilibrium phase was investigated using the linear Langmuir and Freundlich models^73^:

3 $$ \frac{{{\text{C}}_{{\text{e}}} }}{{{\text{q}}_{{\text{e}}} }} = \frac{1}{{{\text{K}}_{{\text{L}}} {\text{Q}}_{{\text{o}}} }} + \left( {\frac{1}{{{\text{Q}}_{{\text{o}}} }}} \right){\text{C}}_{{\text{e}}} $$

4 $$ R_{L} = \frac{1}{{1 + K_{L} C_{o} }} $$

5 $$ \ln {\text{q}}_{{\text{e}}} = \ln {\text{K}}_{{\text{f}}} { } + { }\frac{1}{{\text{n}}}{ }\ln {\text{C}}_{{\text{e }}} $$

*Q*_*o*_ and *K*_*L*_ are the utmost capabilities of the MMT-mAmCs (mg/g) and the adsorption equipoise constant (L/mg). *K*_*F*_ and *n* are the constants relative to the sorption capability of MMT-mAmCs and adsorption strength. These parameters were defined through the slope and intercept of obtained linear figures and then recorded in Table 2. Per correlation coefficients (R^2^) in Fig. S2, the Langmuir isotherm model fitted perfectly with the investigational data obtained (0.993 and 0.998), contrasted with the Freundlich isotherm model which displayed a small R^2^ value. The theoretical maximum adsorption capacities *Q*_*o*_ were 125 and 140.8 mg/g. The *Q*_*o*_ values are near the experimental values of 118 and 137 mg/g of both MB and CV, respectively. Hence, our outcomes suggested that 1 g of used MMT-mAmCs adsorbent can adsorb around 0.125 and 0.141 g of MB and CV from the real wastewater. The obtained data indicated that the K_L_ values are smaller than 1, indicating the reversibility of MB&CV adsorption. Values of 1/*n* are additionally lower than 1, suggesting that the adsorption nature of MB and CV are chemisorption and favorable. The obtained value of R_L_ between 0 and 1 indicates the favorability of the adsorption.

**Table 2:** Langmuir and Freundlich isotherm factors for MB&CV-removal onto MMT-mAmCs under the optimum removal conditions.

	Langmuir		Freundlich	
CV	q_m, exp_ (mg/g)	118		
	R^2^	0.993	R^2^	0.979
	Q_o_ (mg/g)	125	K_F_ (mg^1−(1/n)^ L^1/n^/g)	29.66
	K_L_ (L/mg)	0.307	1/n	0.42
	R_L_	< 1	n	2.38
MB	q_m, exp_ (mg/g)	137		
	R^2^	0.998	R^2^	0.920
	Q_o_ (mg/g)	140.8	K_F_ (L mg^−1^)	60.34
	K_L_ (L/mg)	0.789	1/n	0.26
	R_L_	< 1	n	3.84

In conclusion, the investigation confirmed that MB/CV dyes were adsorbed and formed a monolayer on the homogeneous MMT-mAmCs surface. The adsorbed MB&CV attached to the active sites of MMT-mAmCs through chemical bonds. The great sorption capacity of our MMT-mAmCs adsorbent qualifies it to be a suitable effective adsorbent to remove MB&CV from dyes-rich wastewater.

### Kinetic study of MB/CV-removal

The kinetic study of MB and CV adsorption was explored to define the sorption performance regarding (i) stability with time, (ii) type of MB and CV interaction and binding mechanism with MMT-mAmCs, and (iii) adsorption rate. The removal process of MB and CV was performed at 25 ppm as initial concentration, room temperature, pH 7, and time intervals of 2.5–30 min. The residual MB and CV content was determined by UV–Vis spectrophotometer. The obtained data suggested that for the early 30 min, the sorption was quick because of the abundance of active surface positions. In comparison, the adsorption/removal % became comparatively stable after 30 min, as explained before in the study of stirring time parameter. Therefore, two usually kinetic versions (the pseudo 1st and 2nd order versions) were employed in the following linear forms to fit the obtained results and investigate the possible rate-controlling of MB and CV removal mechanism under optimal removal circumstances. Pseudo 1st order type is generally utilized to discover the sorption behavior in a solid-solution system, while the pseudo 2nd order paradigm is used to evaluate the nature of sorbent-sorbate interaction and a rate-limiting stage^74^:

6 $$ \log \left( {q_{e} - q_{t} } \right) = \log q_{e} - \left( {\frac{{k_{1} }}{2.303}} \right)t $$

7 $$ \frac{{\text{t}}}{{{\text{q}}_{{\text{t}}} }} = \frac{1}{{{\text{k}}_{2} {\text{q}}_{{\text{e}}}^{2} }} + \left( {\frac{1}{{{\text{q}}_{{\text{e}}} }}} \right){\text{t }} $$

*k*_*1*_ and *k*_*2*_ are the 1st and 2nd rate invariable. *q*_*e*_ and *q*_*t*_ are the adsorbed MB&CV amounts at steadiness step and time (*t*). These constants can be determined through the slope and intercept of Log(q_e_-q_t_) and t/q_t_ against time plots. As revealed in Fig. S3 and Table 3, the adsorption of MB and CV dyes fits the pseudo 2nd order, according to R^2^ values. Thus, the adsorption/removal mechanism of MB and CV using MMT-mAmCs is chemically adsorption. This behavior may be due to the rapid binding effect of MB and CV with MMT-mAmCs surface-active sites and the increasing MB&CV transfer/diffusion rate. The rate constant of the MB and CV similarly seems to be controlled by a chemosorption interaction, where the large value of k_2_ also confirms the quicker sorption rate. The linear fitting of the pseudo 1st order is satisfactory (R^2^ ≥ 0.75). Therefore, the achieved q_e_ values are near the experimental adsorption capacity of the MMT-mAmCs for MB&CV dyes.

**Table 3:** Factors of pseudo 1st and 2nd order paradigms for MB&CV removal using MMT-mAmCs adsorbent.

	Pseudo 1st order		Pseudo 2nd order	
CV	R^2^	0.816	R^2^	0.87
	q_e_ mg/g	112.2	q_e_ mg/g	50
	K_1_ min^−1^	0.00691	K_2_ g/mg min	8.8 × 10^−4^
MB	R^2^	0.75	R^2^	0.98
	q_e_ mg/g	125.89	q_e_ mg/g	33.33
	K_1_ min^−1^	0.00506	K_2_ g/mg min	3.7 × 10^−3^

### Thermodynamic studies of MB/CV-removal

**Figure 6:**
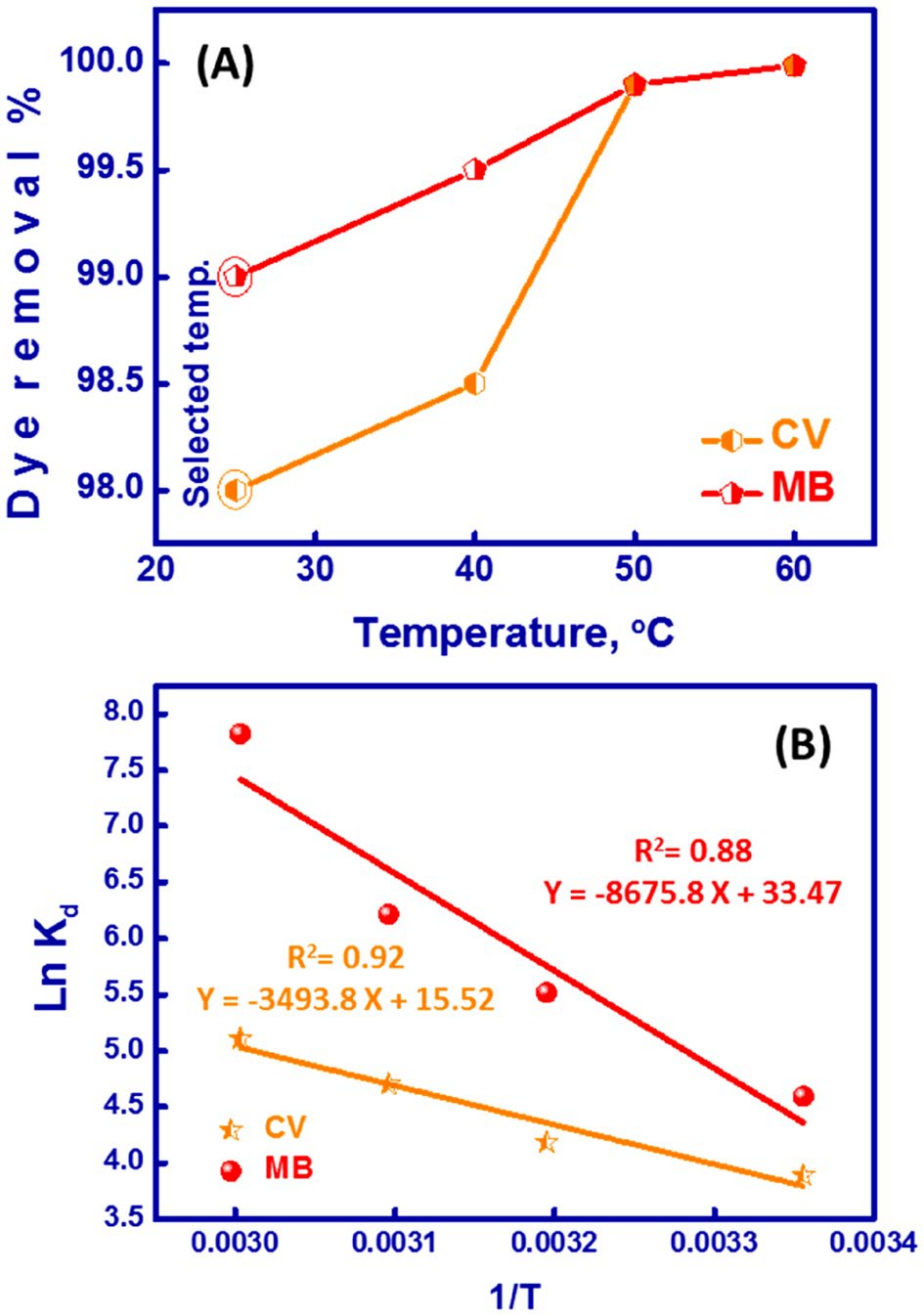
(**A**) Impact of temperature on the MB&CV-removal %, and (**B**) a curve of lnK_d_ vs 1/T to determine of ΔG^°^, ΔS^°^, and ΔH^°^ of MB and CV adsorption using MMT-mAmCs.

Batch trials were performed to investigate temperature’s effect on adsorption and removal of MB and CV dyes. The temperature of the adsorption process can impact the adsorption efficiency via the diffusion rate of MB and CV molecules along with the surface-active sites and into MMT-mAmCs’s pores. The temperature during the MB&CV-removal process gives info regarding variations in enthalpy (ΔH^°^) and entropy (ΔS^°^). MB and CV adsorption showed a slight variation from 98 to 99% at temperatures (25 ± 2 °C to 60 ± 2 °C) using 25 ppm initial concentration and pH 7, as shown in Fig. 6A. In general, expanding temperature may improve the mobility and diffusion of MB and CV molecules due to the formation of surface monolayers. Furthermore, high temperature decreases the intermolecular influences between water and MB&CV molecules, allowing for easier and faster diffusion of the MB and CV into the MMT-mAmCs matrix. Because of the insignificant variation between the removal efficiencies at 25 °C and 60 °C, 25 °C was chosen as an optimal temperature for our study.

To illustrate the thermodynamic parameters and understand the nature of MB&CV adsorption onto MMT-mAmCs at the equilibrium stage, we perform some trials at diverse temperature values ranging from 298 to 333 K. The enthalpy (ΔH^°^), entropy (ΔS^°^), and Gibbs free energy (ΔG^°)^ can be estimated for MB&CV-adsorption onto MMT-mAmCs through the following Equations^75^:

8 $$ lnK_{d} = \frac{{\Delta S^{^\circ } }}{R} - \frac{{\Delta H^{^\circ } }}{RT} $$

9 $$ \Delta G^{^\circ } = \Delta H^{^\circ } - T\Delta S^{^\circ } $$

R is the universal gas constant (8.314 J/mol.k). T is the temperature in kelvin. K_d_ is the equilibrium constant, where K_d _= q_e_/C_e_. Values of ΔH^°^ and ΔS^°^ were defined from a linear plot (ln K_d_ vs. 1/T), as displayed in Fig. 6B. The acquired values of ΔH^°^ and ΔS^°^ are registered in Table 4. The positive value of ΔH^°^ suggested that the MB&CV-adsorption using MMT-mAmCs is an endothermic reaction. The increase in temperature was advantageous by accelerating the adsorption rate and dye diffusion. The negative ΔG^°^ value suggests the spontaneity of the MB&CV-adsorption. Spontaneous MB&CV-adsorption is favorable and preferred. The ΔG^°^ value converted to more negative values with increasing temperature conditions, telling that high temperature was advantageous for MB&CV adsorption. A positive value of ΔS^°^ during MB&CV adsorption onto the MMT-mAmCs shows randomness expansion on the MMT-mAmCs-dye interface, caused by the change of MMT-mAmCs surface or surface movement of adsorbed MB&CV molecules along the adsorbent surface.

**Table 4:** Thermodynamic factors for removing MB and CV dyes onto MMT-mAmCs at various temperatures.

	Temperature K	R^2^	ΔH KJ/mol	ΔS J/mol.K	ΔG KJ/mol
CV	298	0.92	29.047	129.03	9.4
	313				11.3
	323				12.6
	333				13.9
MB	298	0.88	72.13	278.27	10.8
	313				14.9
	323				17.75
	333				20.5

### Regeneration and reusability of MMT-mAmCs

**Figure 7:**
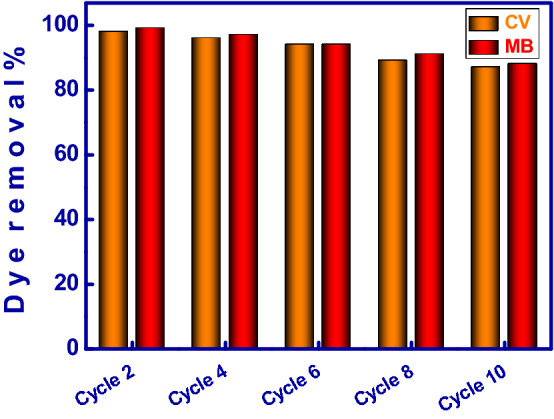
Regeneration and reusability study of the MMT-mAmCs to remove MB and CV dyes.

The proposed MMT-mAmCs should display efficient adsorption efficiency and reusability during numerous adsorption-elution procedures to decrease the full cost of the wastewater treatment operation. The regeneration and reusability of MMT-mAmCs for MB and CV adsorption were explored in ten repeated reuse/cycles (Fig. 7). For every experiment, 50 mg of MMT-mAmCs was stirred with 50 mL of 25 ppm MB/CV solutions at pH 7, and room temperature for 30 min. After the adsorption process, the adsorbed MB and CV onto MMT-mAmCs were eluted using 0.1 mol/L HNO_3_ solutions for 30 min. The treated MMT-mAmCs was washed, filtered, dried at 70 °C, and then used in successive MB and CV adsorption. The gained results signified that the developed adsorbent still retains acceptable adsorption profiles even after reuse for ten repeated adsorption–desorption cycles since the removal efficiency still exceeded 87%. Overall, the results indicated that the MMT-mAmCs is an effective adsorbent in wastewater purification as a consequence of (i) its excellent removal %, (ii) cost-efficiency, (iii) reusability without considerable change in the MMT-mAmCs aptitude even after multiple reuse cycles, and (iv) simple desorption operation of the adsorbed MB and CV dyes at the neutral medium.

### Adsorption of MB and CV from actual water samples

The actual application of the proposed MMT-mAmCs to remove MB and CV from polluted water samples, such as farming wastewater, Nile River water, and industrial wastewater, under optimal removal conditions was explored. In actual application tests, 50 mg of MMT-mAmCs was stirred with 50 mL of water samples at pH 7 and room temperature for 30 min. The farming wastewater, Nile River water, and industrial wastewater samples were filtered through filter paper and centrifuge to eliminate large particles and apparent impurities. The finding results proved that the used MMT-mAmCs adsorbent is considered an efficient adsorbent to remove MB and CV dyes from actual wastewater. Table 5 suggested that the removal % of MMT-mAmCs toward MB&CV dyes was reduced because of further co-existing ions. The primary considerations for the high removal % of MMT-mAmCs may be due to: (i) abundant surface active positions and (ii) rapid and simple diffusion of MB and CV along the MMT-mAmCs surface. Accordingly, the proposed approach can be utilized efficiently to remove MB and CV from actual water to produce healthy water using efficient, non-toxic, and low-priced adsorbent.

**Table 5:** Adsorption of MB and CV from real water samples using MMT-mAmCs.

Water samples	Sample content	Dye spiked (ppm)		Dye adsorption (%)	
		CV	MB	CV	MB
Farming water	Na^+^25.8, k^+^9.94, Ni^2+^0.004, Cd^2+^ 0.003, Zn^2+^0.008, Cu^2+^0.009, Ca^2+^16.3, Mg^2+^5.6, Cr^3+^0.007, Al^3+^0.001, Pb^2+^0.0, Mn^2+^0.001, Hg^2+^0.3106 (ppm)	25	25	95	97.8
Nile River water	k^+^ 14.1, Na^+^ 15.3, Ca^2+^ 8.5, Ni^2+^ 0.14, Cd^2+^ 0.2, Mn^2+^ 0.151, Mg^2+^ 20.3, Cu^2+^ 0.25, Cr^3+^ 0.12, Hg^2+^ 0.251(ppm)	25	25	94.5	97.2
Textile wastewater	k^+^13.5, Na^+^45.5, Mg^2+^16.7, Ca^2+^37.3, Ni^2+^0.01, Mn^2+^0.13, Cu^2+^0.12, Hg^2+^0.96, Cr^3+^0.09, Zn^2+^2.008, Cd^2+^ 0.101, Sr^2+^0.01, Pb^2+^3.0 Al^3+^3.2 (ppm), and other organic pollutants	25	25	91	92.8

## Conclusion

Our investigation provides a sophisticated green adsorbent with remarkable merits, including high adsorption capacity, costless, easy separation, and excellent reusability for removing the cationic MB and CV dyes in a neutral medium. The ZP measurements demonstrated the presence of high negative charges onto the MMT-mAmCs surface reached − 20.5 mV at pH 7, enhancing its ability to grasp the cationic MB and CV from wastewater via the electrostatic interactions. In addition, the experimental study confirmed that the optimal pH to adsorb both cationic dyes was pH 7. These findings elucidated that MMT-mAmCs composite exhibits efficient adsorption processes to MB and CV and a quite simple process that could be implemented in the neutral pH medium and room temperature. Besides, the good magnetic behavior of MMT-mAmCs composite facilitated its separation after the adsorption processes and endowed it with a high ability to recycle and reuse many adsorption cycles. Moreover, the as-fabricated composite showed a promising adsorption behavior since the Q_o_ of MB and CV were 137 and 118 mg/g, respectively. Notably, MMT-mAmCs revealed efficient removal for both MB and CV in the real agricultural water, Nile river water, and wastewater samples at the neutral pH medium.

## Supplementary Information

Supplementary Information.

## Data Availability

The data presented in this study are available on request from the corresponding author.
