# Author Correction: Efficient removal of noxious methylene blue and crystal violet dyes at neutral conditions by reusable montmorillonite/NiFe_2_O_4_@amine-functionalized chitosan composite

**DOI:** 10.1038/s41598-022-25309-9

**Published:** 2022-12-16

**Authors:** Hassanien Gomaa, Eman M. Abd El-Monaem, Abdelazeem S. Eltaweil, Ahmed M. Omer

**Affiliations:** 1 Department of Chemistry, Faculty of Science, Al-Azhar University, Assiut, 71524 Egypt; 2 Chemistry Department, Faculty of Science, Alexandria University, Alexandria, Egypt; 3 Polymer Materials Research Department, Advanced Technology and New Materials Research Institute (ATNMRI), City of Scientific Research and Technological Applications (SRTA-City), New Borg El-Arab City, Alexandria 21934 Egypt

Correction to: *Scientific Reports* https://doi.org/10.1038/s41598-022-19570-1, published online 15 September 2022

The original version of this Article contained an error in Figure 1 labels, where the material names were incorrect.

“mt-mAmCs”.

now reads:

“MMT-mAmCs”.

And “mt”.

now reads:

“MMT”

The original Figure 1 and accompanying legend appear below.

**Figure 1:**
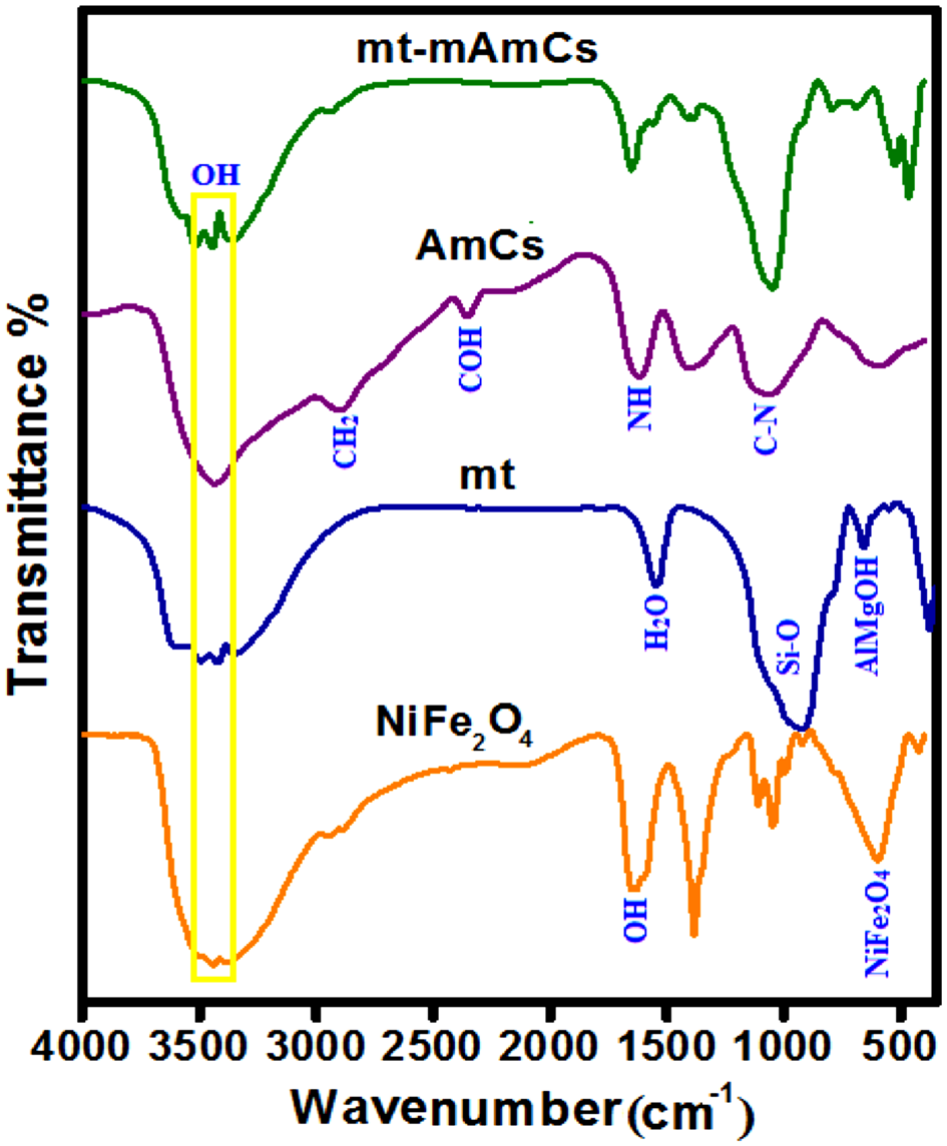
FTIR of NiFe_2_O_4_, MMT, AmCs and MMT-mAmCs composite.

The original Article has been corrected.

