# Supplementary material for: Efficient removal of noxious methylene blue and crystal violet dyes at neutral conditions by reusable montmorillonite/NiFe2O4@amine-functionalized chitosan composite
Source: Sci Rep. 2022 Sep 15;12:15499. doi: 10.1038/s41598-022-19570-1 (PMC9478098; doi:10.1038/s41598-022-19570-1)
Supplement: Supplementary file 1 — Supplementary Information. [file 41598_2022_19570_MOESM1_ESM.docx]

**Efficient removal of noxious methylene blue and crystal violet dyes at neutral conditions by reusable montmorillonite/NiFe_2_O­_4_@amine-functionalized chitosan composite**

**Hassanien Gomaa^1^*, Eman M. Abd El-Monaem^2^*, Abdelazeem S. Eltaweil^2^*, Ahmed M. Omer^3^***

^1^Department of Chemistry, Faculty of Science, Al-Azhar University, Assiut 71524, Egypt.

^2^Chemistry Department, Faculty of Science, Alexandria University, Alexandria, Egypt.

^3^Polymer Materials Research Department, Advanced Technology and New Materials Research Institute (ATNMRI), City of Scientific Research and Technological Applications (SRTA-City), New Borg El-Arab City 21934, Alexandria, Egypt.

***Corresponding authors:** H. Gomaa ([h.gomaa@azhar.edu.eg](mailto:h.gomaa@azhar.edu.eg)), and E. Abd El-Monaem ([emanabdelmonaem5925@yahoo.com](mailto:emanabdelmonaem5925@yahoo.com)), A.S. Eltaweil ([abdelazeemeltaweil@alexu.edu.eg](mailto:abdelazeemeltaweil@alexu.edu.eg)) A.M. Omer ([amomar@srtacity.sci.eg](mailto:amomar@srtacity.sci.eg)).


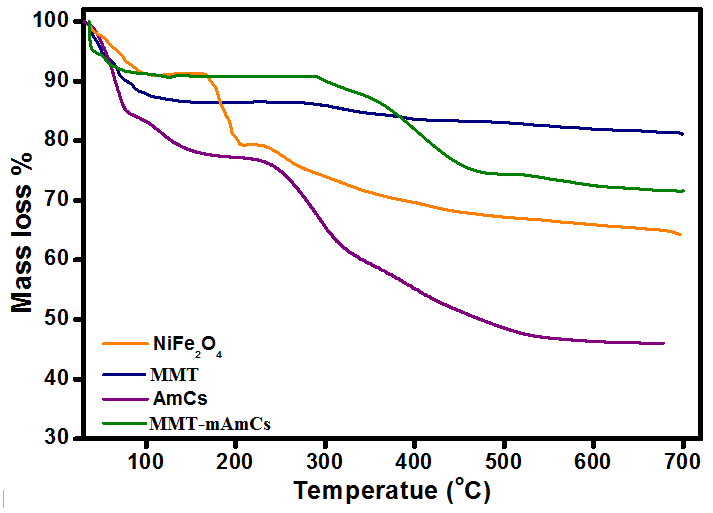


**Fig. S1** TGA of NiFe_2_O_4_, MMT, AmCs and MMT-mAmCs composite


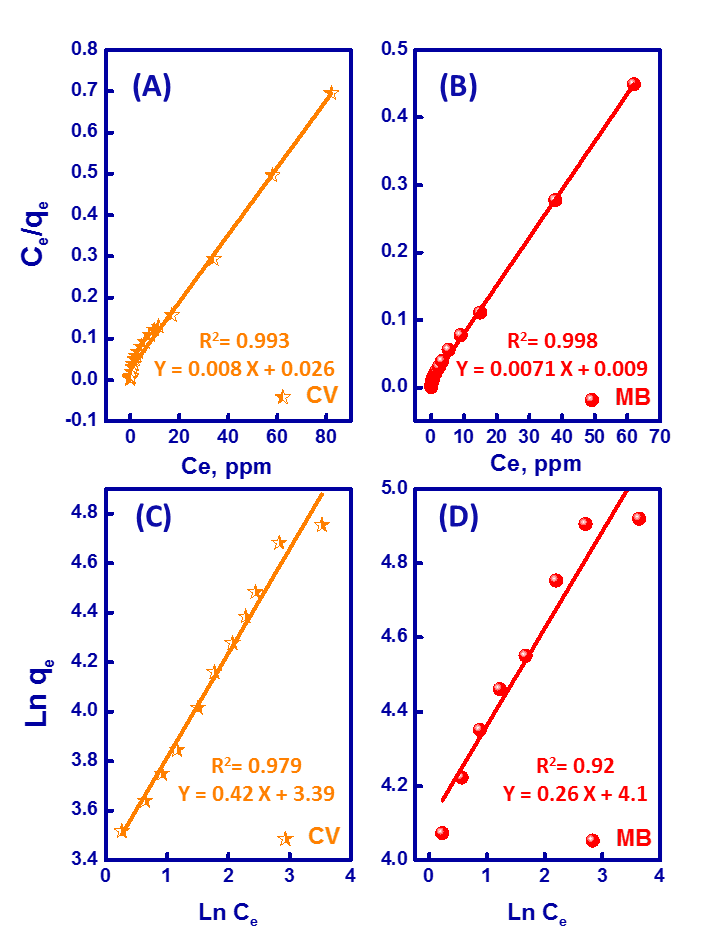


**Fig. S2** The fit-linear diagram of the Langmuir **(A&B)** and Freundlich **(C&D)** isotherm forms for the adsorption of MB and CV using MMT-mAmCs.


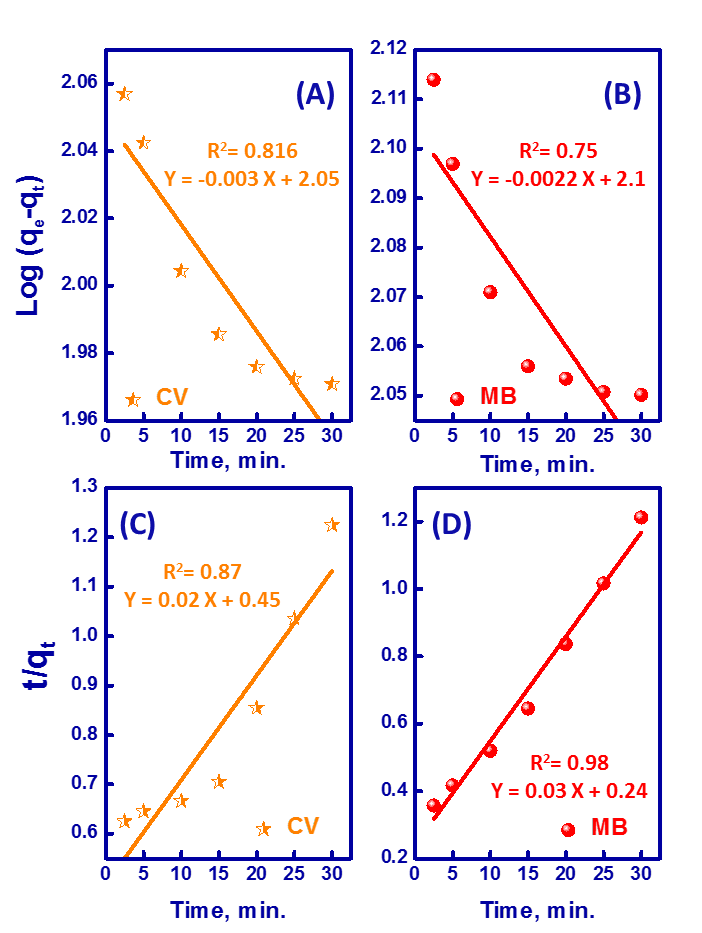


**Fig. S3** The fit-linear diagram of the Pseudo 1^st^ **(A&B)** and 2^nd^ **(C&D)** order kinetic models for the adsorption of MB and CV using MMT-mAmCs.
